# Phonon engineering in proximity enhanced superconductor heterostructures

**DOI:** 10.1038/s41598-017-04057-1

**Published:** 2017-06-27

**Authors:** Yong-Chao Tang, Sangil Kwon, Hamid R. Mohebbi, David G. Cory, Guo-Xing Miao

**Affiliations:** 1 Department of Electrical and Computer Engineering, University of Waterloo, Waterloo, ON N2L 3G1 Canada; 2 Institute for Quantum Computing, University of Waterloo, Waterloo, ON N2L 3G1 Canada; 3 Department of Physics, University of Waterloo, Waterloo, ON N2L 3G1 Canada; 4 Department of Chemistry, University of Waterloo, Waterloo, ON N2L 3G1 Canada; 5 Perimeter Institute for Theoretical Physics, Waterloo, ON N2L 2Y5 Canada; 6 Canada Institute for Advanced Research, Toronto, ON M5G 1Z8 Canada

## Abstract

In this research, we tailor the phonon density of states (DOS) in thin superconducting films to suppress quasiparticle losses. We examine a model system of a proximity-enhanced three-layered Al/Nb/Al heterostructure and show that the local quantized phonon spectrum of the ultrathin Al cladding layers in the heterostructure has a pronounced effect on the superconducting resonator’s quality factors. Instead of a monotonic increase of quality factors with decreasing temperatures, we observe the quality factor reaches a maximum at 1.2 K in 5/50/5 nm Al/Nb/Al microstrip resonators, because of a quantized phonon ladder. The phonon DOS may be engineered to enhance the performance of quantum devices.

## Introduction

With tremendous efforts over the last few decades, superconducting devices emerge as one of the most promising candidates for realizing quantum computation^1–4^. As superconducting circuits scale up, the quality of superconducting thin films plays a more and more important role in determining the ultimate performance of the superconducting quantum networks. Because of the limited choices of superconductor materials for quantum devices, how to improve the existing superconducting materials becomes a major challenge for the further advancement of superconducting quantum information processing. In the Bardeen–Cooper–Schrieffer (BCS) theory, the electron-phonon coupling is the most fundamental properties of superconductors. Adjusting phonon density of states (DOS) can, therefore, tailor the recombination time of Cooper pairs in superconducting thin films and hence affect the coherence time of qubits and quality factors (Q) of resonators. Phonon spectra in superconducting thin films undergo considerable modifications as the phonon wave vectors out of the film plane are restricted by the film thickness, which causes phonon DOS to show discontinuities at specific energies. To the best of our knowledge, phonon engineering has not been realized in superconducting resonators since it can only occur in ultra-thin films which will induce significant microwave losses for resonators and decoherence for qubits. In a trilayer heterostructure consisting of one thick core layer and two thin cladding layers, the phonon quantization shows up in the cladding layers due to the local phonon DOS. Such superconductor heterostructure can be readily utilized for improving quantum devices.

In this paper, we examine a model system of proximity-enhanced Al/Nb/Al heterostructures, and we consider the size effect of the ultrathin Al cladding layers. The two Al layers are thin enough such that the size effect of thin films becomes appreciable. For thin enough films, the DOS shows discrete steps instead of the smooth curve (∝Ω^2^, where Omega is the phonon frequency) under a low-frequency approximation. When a phonon DOS step coincides with the superconducting gap edge, the size effect manifests as an anomalous peak of Q before it levels off with decreasing temperature. The Q reaches an unusual maximum because the inverse of quasiparticle lifetime is proportional to the phonon DOS in the low-temperature approximation^5^. The reduction of phonon DOS below certain energies (before each jump on the phonon spectrum) may be utilized to reduce quasiparticles decay rate in thin Al layers for quantum circuits^6, 7^.

Furthermore, the proximity effect will enhance the performance of resonators under magnetic fields, for example, for pulsed electron spin resonance (pulsed-ESR) based quantum computing^8^. For our targeted pulsed-ESR experiments, the required magnetic field is around 0.35 T, corresponding to a g = 2 electron resonant frequency of 9.8 GHz, and the field drastically reduces the quality factor of superconducting resonators. Thus, a higher Q of a resonator under such a strong magnetic field can be reached, better performance for quantum information processing is desired. Q will be limited by magnetic flux trapped in thin films in the form of vortices^9^ which physically oscillate in microwave driving currents and induce losses. Vortex starts to be generated in Al thin film when a magnetic field is greater than a threshold *B*_*th*_ (for example, *B*_*th*_ = 0.3 Gauss for 150 nm Al^10^). In our Al/Nb/Al trilayer structures, the Nb core layer can enhance the critical field and critical temperature of Al films through the proximity effect as Nb^11^ is significantly higher on both parameters. Due to the skin effect of microwave propagation, the amplitude of the electromagnetic waves will decay exponentially inside the superconductors with a length scale of the penetration depth. So the main contribution to the microwave losses comes from the surfaces of the superconducting films as the significant amount of currents flow here^12^. Proximity effect will induce higher critical field, thereby suppressing the generation of vortices in the Al layers and reduce losses on the surfaces; also, higher induced critical temperatures exponentially reduce the population of quasiparticles, which also reduces losses in these layers.

This paper is organized as follows: A model of Q from the trilayer Al/Nb/Al resonators is introduced first, which considers the size effect of the thin cladding Al layers. The measured Q in 5/50/5 nm and 10/50/10 nm Al/Nb/Al heterostructures are compared in the following section. The Q from the 5/50/5 nm heterostructure has a clear peak at 1.2 K, which is due to the reduced surface losses from suppressed phonon DOS in the thin Al layers. The physical growth of Al/Nb/Al heterostructure and their structural characterization are discussed at last.

## Results and Discussion

### Microwave losses induced by quasiparticle-lifetime broadening

**Figure 1:**
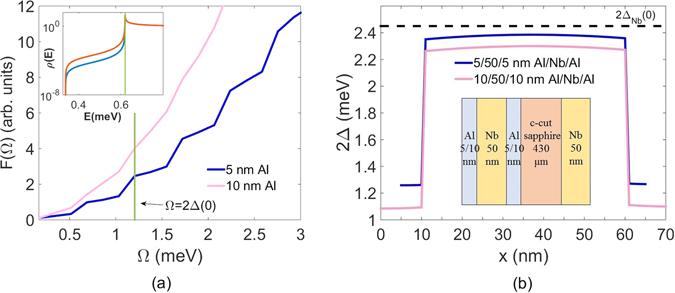
(**a**) Calculated total phonon density of states as a function of phonon energy for 5 nm and 10 nm Al films. The vertical line shows where the gap energy 2Δ(0) for 5 nm Al in the heterostructure is located. The inset illustrates how quasiparticle DOS at T = 1.2 K broadens when *θ* varies from 10^−4^ (cyan curve) to 6 × 10^−4^ (red curve). The vertical line shows where the quasiparticle energy equaling to Δ(0) for 5 nm Al in the heterostructure is located. (**b**) Calculated superconducting pair potential distribution with depth for 5/50/5 and 10/50/10 nm Al/Nb/Al heterostructure. The dashed line is the gap energy for a single 50 nm Nb layer determined from our experiment. The inset is the schematic illustration of the Al/Nb/Al heterostructure on a double-side-polished c-cut sapphire. The back side is coated with 50 nm Nb.

To employ the suppressed phonon DOS in proximity enhanced Al/Nb/Al trilayer films, we build a model to show how the local phonon DOS in the thin Al layers could affect the resonator Q of the whole structure. The Q values may be affected by quasiparticles losses which are induced by the smearing of quasiparticles states at the gap energy edge. The response of a superconductor, when driven away from equilibrium in microwave frequency, is dependent upon the various electron relaxation times. Central to the relaxation phenomenon is the recombination time *τ*_*R*_. In order to explain the lifetime-broadened energy gap edge, Dynes *et al*. proposed to add a parameter −*j*Γ_*D*_ ($j=\sqrt{-1}$) to quasiparticle energy *E* in the formula of excitation spectrum given by BCS theory $\rho (E,{{\rm{\Gamma }}}_{D})={\rm{Re}}\{(E-j{{\rm{\Gamma }}}_{D})/{[{(E-j{{\rm{\Gamma }}}_{D})}^{2}-{{\rm{\Delta }}}^{2}]}^{\mathrm{1/2}}\}$ ^13^. It was justified by Kaplan *et al*. that the parameter 2Γ_*D*_ could be interpreted as the inverse of the quasiparticle recombination lifetime^5^. Mitrovic *et al*. proposed an alternative form of the excitation spectrum by including a complex gap energy Δ = Δ_0_ − *j*Δ_2_ from the first principles^14^. Indeed, *ρ*(*E*, Γ) is replaced by $\rho (E,{{\rm{\Delta }}}_{2})={\rm{Re}}\{E/[{E}^{2}-{({{\rm{\Delta }}}_{0}-j{{\rm{\Delta }}}_{2})}^{2}{]}^{\mathrm{1/2}}\}$ and $-2\,{\rm{Im}}\,{\rm{\Delta }}({\bf{k}},E={{\rm{\Delta }}}_{0})$ is equal to the inverse quasiparticle lifetime with **k** on the Fermi surface. It is well known that the imaginary part of the gap energy is nonzero at a finite temperature as a result of quasiparticle damping^15^. Per the modified formula for excitation spectrum, if Δ_2_ becomes larger, there will be an increase in the gap smearing as shown in the inset of Fig. 1(a). Defining $\theta \equiv {{\rm{\Delta }}}_{2}/{{\rm{\Delta }}}_{0}$, we plot the quasiparticle DOS at T = 0 K for *θ*_1_ = 6 × 10^−4^ and *θ*_2_ = 10^−4^ from the upper curve to the lower curve in the inset, respectively. The values of *θ* are chosen for the purpose of demonstrating the effect of a complex gap energy on the edge smearing. Thus, there are more density of states for quasiparticles inside the gap. The total density of quasiparticles near the gap will be increased, causing more losses.

#### Quasiparticle decay rate

The decay rate 2Γ(*ω*) of a quasiparticle with energy *ω* is equal to 2Γ_*s*_(*ω*) + 2Γ_*r*_(*ω*), where 2Γ_*s*_(*ω*) corresponds to quasiparticle scattering processes with the emission or absorption of a phonon, and 2Γ_*r*_(*ω*) is the recombination rate corresponding to a process in which one quasiparticle recombines with another to form a Cooper pair with the excess energy emitted as a phonon. For quasiparticle scattering processes at low temperatures, the important phonon energies are near zero, not 2Δ(0), so the change of phonon DOS for phonons with energies at gap edge has no impact on the scattering rate. On the other hand, according to the Eliashberg formulation^16^, the expression for 2Γ_*r*_(*ω*) is ref. 5:

1 $$\begin{array}{lll}2{{\rm{\Gamma }}}_{r}(\omega ,T) & \propto  & \frac{1}{1-f(\omega )}{\int }_{\omega +{\rm{\Delta }}}^{\infty }\,d{\rm{\Omega }}{\alpha }^{2}({\rm{\Omega }})F({\rm{\Omega }}){\rm{Re}}(\frac{{\rm{\Omega }}-\omega }{{[({\rm{\Omega }}-\omega {)}^{2}-{{\rm{\Delta }}}^{2}]}^{\mathrm{1/2}}})\\  &  & \times (1+\frac{{{\rm{\Delta }}}^{2}}{\omega ({\rm{\Omega }}-\omega )})[n({\rm{\Omega }})+1]\,f({\rm{\Omega }}-\omega ),\end{array}$$

where Ω is the energy of phonon, 2Γ_*r*_(*ω*) is related to the low-frequency part of the phonon DOS *F*(Ω) weighted by the square of the matrix element of the electron-phonon interactions *α* ^2^(Ω), *f*(*ω*) and *n*(Ω) are the state occupations for quasiparticles and phonons, respectively. As was pointed out by Martinis *et al*.^7^, non-equilibrium quasiparticles are expected in a superconductor well below the critical temperature and result in finite state occupation *f*(*ω*) of quasiparticles even at very low temperatures. By assuming *α* ^2^(Ω) to be constant in the vicinity of Ω = 2Δ(*T* = 0) for Al^5^, the transition probability for this relaxation process should be nonzero at any finite temperature and is directly proportional to the phonon DOS at the gap edge.

#### Local phonon DOS

The local phonon DOS in a thin metal film backed by a semi-infinite metal substrate is not much different from that of an isolated metal film, except for some smearing on sharp structures^17^. In our case, the core Nb layer is indeed much thicker and heavier than the cladding Al layers. Thus we can approximate the local DOS of the Al coating layers with that of isolated thin Al films. In a thin film, the phonon DOS gains discrete steps at characteristic energies^18^. Here we adopt the equation of motion for elastic vibrations in an anisotropic medium to calculate the local phonon DOS in the Al cladding layer^19^,

2 $$\rho \frac{{\partial }^{2}{U}_{m}}{\partial {t}^{2}}=\frac{\partial {\sigma }_{mi}}{\partial {x}_{i}}(m,i=1,2,3),$$

where $\overrightarrow{U}({U}_{1},{U}_{2},{U}_{3})$ is the displacement vector, *ρ* is the mass density of the material, *σ*_*mi*_ is the elastic stress tensor given by *σ*_*mi*_ = *c*_*mikj*_ *U*_*kj*_. *c*_*mikj*_ is the elastic module, and ${U}_{kj}=\frac{1}{2}(\frac{\partial {U}_{k}}{\partial {x}_{j}}+\frac{\partial {U}_{j}}{\partial {x}_{k}})$ is the strain tensor. The total phonon DOS is obtained by summing over all phonon modes, $F({\rm{\Omega }})=\frac{1}{2\pi }\sum _{n}\,{k}_{n}({\rm{\Omega }})\frac{d{k}_{n}({\rm{\Omega }})}{d{\rm{\Omega }}}$ (*n* is the index of phonon modes). The material parameters used in our calculations are taken from the literature^20^, and the calculations have considered that the Al layers in our actual heterostructure are oriented in the (111) direction.

Figure 1(a) shows the calculated phonon DOS for 5 nm and 10 nm Al thin films. In the 10 nm Al film, the phonon DOS shows a nearly smooth dispersion curve, which leads to a gradual increase in losses. On the other hand, the 5 nm Al film clearly shows noticeable jumps in the phonon spectra due to the strong size effect. The inset in Fig. 1(a) illustrates how the increasing theta can increase the subgap quasiparticle DOS. The imaginary part is increased from T = 1.2 K to T = 0.2 K. Because of a larger imaginary part of gap energy, there exist more quasiparticle DOS inside the gap which cause more losses at the lower temperature.

The pair potential defines the strength of BCS pairing interaction and is spatially dependent. Since the density of states in Al and Nb is modified due to the proximity effect^21, 22^, the pair potential experiences strong variations along thickness. Though the pair potential at Al layer is lifted while the pair potential at Nb layer is lowered by proximity effect, it has a discontinuity at the interface between Al and Nb because the physical presence of interfaces ensures the presence of scattering centers. To clarify the effect of the local phonon DOS on quasiparticle induced losses, we calculate the local pair potential inside the 5 nm cladding Al film from the Usadel equations^23, 24^. The interface parameters between Al and Nb are assumed with literature values^25^. If the interfaces affect several atomic layers of both superconductors, the boundary conditions make the position dependence of pair potential look like steps as in Fig. 1(b). The pair potential is 1.3 meV for 5 nm Al and 1.1 meV for 10 nm Al in the trilayer structure, which are close to the calculated values reported by Brammertz *et al*.^24^ for a similar Al/Nb/Ta structure. The gap energy 1.3 meV matches the second phonon DOS jump in Fig. 1(a). When the pair potential increases beyond the first phonon DOS jump with decreasing temperatures, the effect of sudden increased phonon DOS is shadowed by the fast reduction of quasiparticle population. As the quasiparticle population begins to level off at lower temperatures, the second sudden jump of phonon DOS will result in a fast increase in losses when the gap edge falls upon the phonon steps. Therefore, by adjusting the thickness of the cladding Al layers, we can observe phonon induced Q variation with temperature.

### Resonator measurement

**Figure 2:**
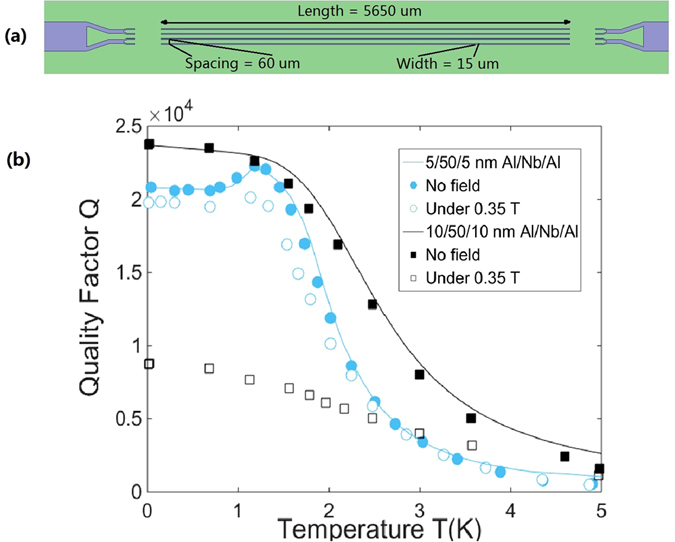
(**a**) Layout of the resonator device. It consists of four parallel *λ*/2 microstrip line resonators separated by 60 *μm*. Each *λ*/2-resonator is 5650 *μm* long, 15 *μm* wide. (**b**) The Q values of 5/50/5 nm and 10/50/10 nm Al/Nb/Al trilayer structures. The filled circles and squares are measured values for 5/50/5 and 10/50/10 Al/Nb/Al resonators without external fields; the empty circles and squares are measured values under a 0.35 T in-plane field. The solid lines are fitted curves from our model.

We used optical lithography and reactive ion etching to fabricate the composite half-wavelength microstrip transmission line resonators^26^ from these trilayer structures, as shown in Fig. 2(a). Compared to 3D cavities, or coplanar waveguides (CPW), microstrip line resonators offer small mode volume and can produce homogeneous magnetic fields above the resonator at microwave frequency, and therefore are better suited for pulsed ESR based quantum circuits.

The resonator measurement was carried out in a closed-flow dilution refrigerator (Leiden Cryogenics BV, BA Leiden, Netherlands) with a vector network analyzer (Agilent N5230A) to record the transmission through our feedline, *S*_21_. Every data point was obtained by averaging over measurements. The resulting Q as a function of temperature is presented in Fig. 2(b). The filled circles and squares represent the Q for 5/50/5 nm and 10/50/10 nm Al/Nb/Al resonators, respectively, without external magnetic field. Both films show similar Q of 22000 at 1.2 K, and it is also the highest Q for the 5/50/5 nm resonator. The two samples behave very differently as the temperature lowers further. The resonator with 5 nm Al cladding layers has its Q slowly dropped to 20800 at 30 mK, while the other one shows gradually increased Q. The different behavior between 5/50/5 nm and 10/50/10 nm resonators can be explained by the size effect of the Al cladding layers. From the earlier discussions, the proximity-enhanced energy gap of the 5 nm Al layers is estimated to be $2{\rm{\Delta }}\mathrm{(0)}\approx 1.3\,{\rm{meV}}$. It happens to be right above the second phonon step as shown in Fig. 1(a). As the temperature rises from 0 K, the gap gradually narrows. At a temperature T (1.2 K in this case), the gap edge moves below the phonon step, and the phonon density dropped significantly leading to reduced loss and improved Q. As a comparison, the size effect for 10 nm Al layers is negligible. Therefore, the 10/50/10 nm Al/Nb/Al resonator shows continuously decreasing Q as the temperature goes up, while the 5/50/5 nm resonator has an anomalously increased Q around 1.2 K. It is noted that Martinis *et al*.^7^ observed an improvement in quasiparticle damping when tuning the temperature for superconducting qubits. The unusual reduction in damping rate is explained with the redistribution of the state occupation *f*(*ω*) for non-equilibrium quasiparticles as temperature decreases. In our experiments, 5/50/5 nm and 10/50/10 nm Al/Nb/Al trilayer films have almost same gap energy and are grown on the same kind of substrates. The resonators with same design were also measured in the same environment. We can further deduce from the Fig. 2 that the devices suffer from the same non-equilibrium quasiparticle density as the Q values begin to level off at the same temperature T = 1.2 K. Only by the changes in *f*(*ω*) cannot the experimental results for 10/50/10 nm and 5/50/5 nm resonators be explained at the same time. Indeed, both of *f*(*ω*) and *F*(Ω) will affect quasiparticle damping rate. Thus, they may cause the same unusual peak as observed in experiments.

We can more quantitatively model the Q in our resonators with surface impedance calculations^27^. The trilayer structure can be treated as the cascade of three single-layer films, and the total surface impedance can be evaluated by concatenating the surface impedance of each layer through the traditional transmission line theory^28^. The solid lines shown in Fig. 2(b) are the best fit obtained with this model. Without external magnetic fields applied, resonators with Al cladding showed clearly enhanced performance over resonators with only 50 nm Nb fabricated in the same way (Q $\approx$ 15000). Pure Al does have better resonator performance than pure Nb in zero fields, and our Al cladding allows a significant portion of microwaves to transport in the surface Al layers instead of the core Nb layer.

When an in-plane magnetic field of *B*_0_ = 0.35 T is applied in parallel to the resonator lines, both resonators show lower Q than in the zero-field situation because of weakened pairing. For the 10/50/10 Al/Nb/Al sample, maximum Q significantly drops from 23800 to 8800. The proximity effect between Nb and Al may not be robust enough to enhance the 10 nm Al layers entirely, and thicker Al layers are also more susceptible to vortices formation. Therefore, it performs worse than the device with thinner Al cladding layers. The 5/10/5 Al/Nb/Al device maintains Q as high as 19800 at 15 mK and the maximum Q = 20100 at 1.2 K when a *B* = 0.35 T field is applied. Compared with the case of zero magnetic fields, the Q is only moderately decreased because of a small reduced-field (for 50 nm Nb thin film, the reduced-field is ${B}_{0}/{B}_{c2}\mathrm{(0)}\approx 0.1$ ^29^). The peak Q at 1.2 K drops slightly larger than the Q at lowest temperatures. This is due to the losses generated by the movement of quasiparticles at a higher temperature (>1 K). Nevertheless, this result has surpassed the previously reported best results of 6000 on 400 nm Nb resonators^26^ and 15000 on 50 nm Nb resonators under the same magnetic field with the same resonator design.

In this work, we identified the effects of local phonon DOS on the performance of the proximity-enhanced Al/Nb/Al trilayer resonators. We modeled and observed an unusual increase in Q with temperature when the superconductor gap edge moves below a phonon DOS step. And Q values higher than 20000 were achieved under a 0.35 T in-plane magnetic field, making them suitable for operations when magnetic fields are necessary.

## Methods

### Model for surface impedance of trilayer structure

The microwave loss of superconducting resonators or transmission lines can be explained by resistive loss from surface impedance^30, 31^. Surface impedance reveals the input impedance per unit area of the whole structure toward the direction of wave propagation. The quality factor is defined as $Q\propto {X}_{s}/{R}_{s}$ (*X*_*s*_ and *R*_*s*_ are the total reactance and the resistive component of the surface impedance, respectively). Because the field analysis of parallel propagation of a wave along the conductor yields the same solutions and surface impedance as that of the normal incidence scenario^32^, transmission lines where the electric field travels along it can be modeled as that a TEM wave is normally incident upon the conductor. This also applies to trilayer structures. Therefore, the surface impedance of the microstrip superconducting trilayer resonator is the same as that of the perpendicular incidence scenario. In this case, the multi layers can be treated as the cascade of many single-layer films. The surface impedance *Z*_*m*_ for the *m*_th_ superconducting layer after (*m* − 1)_th_ layer can be evaluated through^28^:

3 $${Z}_{m}=\frac{{\gamma }_{m}}{{\sigma }_{m}}\frac{{{\rm{e}}}^{{\gamma }_{m}{t}_{m}}+\frac{{\sigma }_{m}\,{Z}_{m-1}-{\gamma }_{m}}{{\sigma }_{m}\,{Z}_{m-1}+{\gamma }_{m}}{{\rm{e}}}^{-{\gamma }_{m}{t}_{m}}}{{{\rm{e}}}^{{\gamma }_{m}{t}_{m}}-\frac{{\sigma }_{m}\,{Z}_{m-1}-{\gamma }_{m}}{{\sigma }_{m}\,{Z}_{m-1}+{\gamma }_{m}}{{\rm{e}}}^{-{\gamma }_{m}{t}_{m}}}={R}_{m}+j{X}_{m},$$

where *t*_*m*_ is the *m*_*th*_ layer thickness, *γ*_*m*_ = (1 + *j*)/*λ*_*m*_ is the complex propagation constant of the *m*_th_ layer, *λ*_*m*_ is the London penetration depth of the layer, *σ*_*m*_ is the complex superconducting conductivity for the frequency *ω* and can be calculated by the extended Zimmermann formula, *μ* is the permeability of the layer, *Z*_*m*−1_ is the impedance of the load which yields the input impedance of the total layers before the layer, and equals to 377 Ω which is the characteristic impedance of free space when the layer is exposed in air.

To calculate the complex conductivity, *σ*_*s*_(*ω*) = *σ*_1_ − *jσ*_2_, for a layer of superconductor with arbitrary purity, the Zimmermann formula^33^ can offer an easy method when arbitrary mean free path length is considered. Zimmermann expression is generalized for the conductivity of a homogeneous isotropic BCS superconductor with complex gap energy which can be applied to the samples with any purity^27^. The explicit expression for the conjugate of the complex conductivity ${\sigma }_{s}^{\ast }(\omega )={\sigma }_{1}+j{\sigma }_{2}$ with Δ = Δ_0_ − *j*Δ_2_ reads:

4 $$\frac{{\sigma }_{1}+j{\sigma }_{2}}{{\sigma }_{n}}=\frac{j}{2\omega \tau }\times ({\int }_{{\rm{\Delta }}}^{\hslash \omega +{\rm{\Delta }}}\,{{\rm{I}}}_{1}dE+{\int }_{{\rm{\Delta }}}^{\infty }\,{{\rm{I}}}_{2}dE),$$

5 $$\begin{array}{rcl}{I}_{1} & = & \tanh \,\frac{E}{2kT}\{[1-\frac{{{\rm{\Delta }}}^{2}+E(E-\hslash \omega )}{{P}_{3}{P}_{2}}]\frac{1}{{P}_{3}+{P}_{2}+j\hslash /\tau }\\  &  & -[1+\frac{{{\rm{\Delta }}}^{2}+E(E-\hslash \omega )}{{P}_{3}{P}_{2}}]\frac{1}{{P}_{3}-{P}_{2}+j\hslash /\tau }\},\end{array}$$

6 $$\begin{array}{rcl}{I}_{2} & = & \tanh \,\frac{E+\hslash \omega }{2kT}\{[1+\frac{{{\rm{\Delta }}}^{2}+E(E+\hslash \omega )}{{P}_{1}{P}_{2}}]\frac{1}{{P}_{1}-{P}_{2}+j\hslash /\tau }\\  &  & -[1-\frac{{{\rm{\Delta }}}^{2}+E(E+\hslash \omega )}{{P}_{1}{P}_{2}}]\frac{1}{-{P}_{1}-{P}_{2}+j\hslash /\tau }\}\\  &  & +\,\tanh \,\frac{E}{2kT}\{[1-\frac{{{\rm{\Delta }}}^{2}+E(E+\hslash \omega )}{{P}_{1}{P}_{2}}]\frac{1}{{P}_{1}+{P}_{2}+j\hslash /\tau }\\  &  & -[1+\frac{{{\rm{\Delta }}}^{2}+E(E+\hslash \omega )}{{P}_{1}{P}_{2}}]\frac{1}{{P}_{1}-{P}_{2}+j\hslash /\tau }\},\end{array}$$

where ${P}_{1}=\sqrt{{(E+\hslash \omega )}^{2}-{{\rm{\Delta }}}^{2}}$, ${P}_{2}=\sqrt{{E}^{2}-{{\rm{\Delta }}}^{2}}$, ${P}_{3}=\sqrt{{(E-\hslash \omega )}^{2}-{{\rm{\Delta }}}^{2}}$, *σ*_*n*_ is the normal conductivity of the superconductor before the superconducting transition happens. *τ* is the electron collision time at Fermi surface in normal state and is subject to film variance. The complex gap energy Δ in Eqs (5) and (6) is calculated by Eq. (1). *f*(*ω*) is the sum of thermal occupation and an exponentially decaying occupation due to the non-equilibrium quasiparticles, $f(\omega )=1/\mathrm{(1}+{e}^{\omega /T})+1/\mathrm{(1}+{e}^{\omega /{T}_{0}})$, where *T*_0_ is a fitting parameter. The total state occupation for quasiparticles is then finite at zero temperature, as discussed by Martinis *et al*.^7^.

### Film characterization

The structure of the composite film is based on Al/Nb/Al trilayer, as shown in the inset of Fig. 1(b). The films were deposited on the front side of a double-side-polished sapphire wafer, while the back side of the wafer was coated with 50 nm pure Nb as the ground plane of the superconducting transmission line resonators. Pure Nb, deposited at room temperature without pre- and post-annealing, was chosen as the ground plane to avoid heating damage to the materials already present on the front side. The samples were deposited in a reactive magnetron sputtering system (AJA International, USA) with the base pressure of 1 × 10^−8^ Torr. The substrates were first Ar-sputter cleaned for 5 minutes under substrate bias with 50 W RF power, 5 milli-Torr chamber pressure and 30 sccm of Argon flow. Then they were annealed at 700 °C for 1 hour and cooled to room temperature before the actual film growth starts.

**Figure 3:**
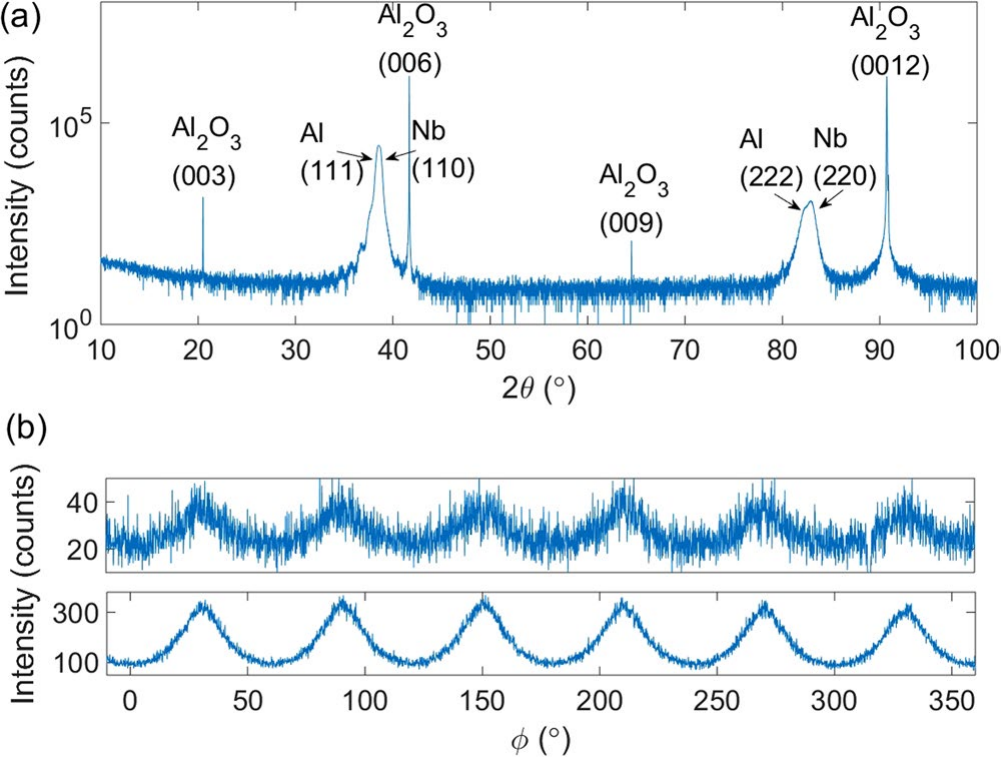
(**a**) High-resolution XRD pattern of the Al/Nb/Al trilayer film on the c-cut sapphire. (**b**) Azimuthal XRD data (“*ϕ*-scan”) reveals the six-fold symmetry of the off-axis (200) reflections from Al and Nb, respectively.

X-ray diffraction (XRD) was used to study the crystal structures of the composite films grown on the c-cut sapphire. The measurement was carried out using a Bruker D8 Discover (Bruker AXS, Germany) system with a Cu *K*_*α*_ source. Figure 3(a) shows the high-resolution 2*θ* − *ω* scan of the composite film. A 3-bounce monochromator was used in front of the source in addition to a Gobel mirror. Besides four extremely sharp peaks from the sapphire substrate, there are only peaks from Al (111) at 38.47°, Nb (110) at 38.51° and their second order diffractions. Al (111) and Nb (110) are so close that they cannot be well distinguished from each other, but the Al (222) and Nb (220) clearly revealed the presence of both peaks. The appearance of only Al 111 and Nb 110 peaks indicate that Al and Nb are both textured. Figure 3(b) is the azimuthal XRD data (“*ϕ*-scan”) from the Al/Nb/Al composite film. The upper panel is the *ϕ*-scan for Al (200) by setting *ψ* at 54.7° – the angle between the (111) plane and (200) plane in an fcc structure. The detection angle 2*θ* was set at 44.74° which is for Al (200) reflections. Fully epitaxial Al (111) should display three-fold symmetry in the *ϕ*-scan of (200) planes. The appearance of six peaks indicates two symmetrically equivalent growth orientations (twins) occur in the Al layers, as a result of stacking three-fold symmetric fcc structures onto the six-fold symmetric hcp substrate^34^. The lower panel of Fig. 3(b) is the *ϕ*-scan for Nb (200) by setting *ψ* at 45°. The six-fold symmetry from Nb (200) can also be attributed to the twin growth of the bottom Al layer. We also performed 2*θ* − *ω* scans by fixing *ϕ* at one of the off-axis peaks, such as 30° in Fig. 3(b). The Bragg diffractions confirmed the *ϕ* peaks come from 2*θ* of 44.7° for Al and 55.5° for Nb, well matching the Al (200) and Nb (200) reflections. It proves that the six-fold symmetry shown in Fig. 3(b) indeed originates from the off-axis diffractions from Al (200) and Nb (200) planes.
